# Local Circulation of Sindbis Virus in Wild Birds and Horses, the Netherlands, 2021–2022

**DOI:** 10.3201/eid3104.241503

**Published:** 2025-04

**Authors:** Kiki Streng, Cora M. Holicki, Jenny C. Hesson, Heather Graham, Felicity Chandler, Louie Krol, Rody Blom, Emmanuelle Münger, Anne van der Linden, Constantianus J.M. Koenraadt, Maarten Schrama, Chiara de Bellegarde de Saint Lary, Leo G. Visser, Bas Oude Munnink, Åke Lundkvist, Marion P.G. Koopmans, Henk P. van der Jeugd, Wim H.M. van der Poel, Reina S. Sikkema

**Affiliations:** Wageningen University & Research, Wageningen, the Netherlands (K. Streng, R. Blom, C.J.M. Koenraadt, W.H.M. van der Poel); Erasmus Medical Center, Rotterdam, the Netherlands (C.M. Holicki, F. Chandler, E. Münger, A. van der Linden, B. Oude Munnink, M.P.G. Koopmans, R.S. Sikkema); Nedre Dalälvens Utvecklings AB, Gysinge, Sweden (J.C. Hesson); Uppsala University, Uppsala, Sweden (J.C. Hesson, Å. Lundkvist); Wageningen Bioveterinary Research, Lelystad, the Netherlands (H. Graham, W.H.M. van der Poel); Institute of Environmental Sciences, Leiden University, Leiden, the Netherlands (L. Krol, M. Schrama); Julius Centre for Health Sciences and Primary Care, University Medical Center, Utrecht, the Netherlands (C. de Bellegarde de Saint Lary); Leiden University Medical Center, Leiden University Center for Infectious Diseases, Leiden (C. de Bellegarde de Saint Lary, L.G. Visser); Vogeltrekstation—Dutch Centre for Avian Migration and Demography, Netherlands Institute of Ecology, Wageningen (H.P. van der Jeugd)

**Keywords:** Sindbis virus, viruses, zoonoses, horses, birds, mosquitoes, vector-borne infections, epidemiologic surveillance, the Netherlands

## Abstract

We report Sindbis virus circulation in the Netherlands based on serologic evidence found in 6 resident wild birds and 3 horses (2021–2022). Tested mosquitoes were molecularly negative, and humans were serologically negative. Veterinarians and health practitioners in the Netherlands should be aware of the importance of surveillance for Sindbis virus.

Sindbis virus (SINV; family Togaviridae, genus *Alphavirus*) is maintained in an enzootic transmission cycle between birds (e.g., passerines and grouse) and mosquito vectors (mainly *Culex* spp., but also *Aedes* and *Culiseta* spp.) (*1*). Horses and humans are considered dead-end hosts. Clinical cases in humans are commonly reported in northern Europe (Finland and Sweden) and South Africa (*2*,*3*). Studies have described nonsymptomatic infections in horses in Sweden through the detection of neutralizing antibodies (*4*), but neurologic signs can also develop in horses, as shown in South Africa (*5*). Many countries in Europe have reported evidence of exposure in wildlife, including Germany and the United Kingdom, but not the Netherlands (*2*). Therefore, we investigated the presence of SINV in the Netherlands in mosquitoes, birds, horses, and humans.

Throughout the Netherlands, we screened mosquitoes, wild birds, horses, and humans for SINV RNA and neutralizing antibodies (Figure; Appendix Figure 1). We sampled mosquitoes (n = 12,884) from July to mid-October in 2020, 2021, and 2022 as previously described (*6*) and live wild birds (n = 10,983) in 2021 and 2022 as part of ongoing research into the arbovirus dynamics in birds (*7*). We collected serum samples from horses (n = 368) from May 2021 through 2022 (*8*) and additional serum samples (n = 3) in October 2023. We also collected serum samples from bird ringers (n = 148), which are actively ringing birds and may be at higher risk for infection, from June to September 2021 as previously described (*9*).

**Figure:**
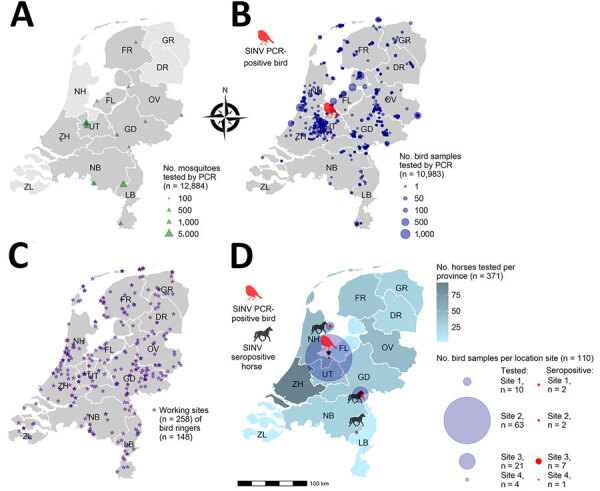
Local circulation of SINV in wild birds and horses, the Netherlands, 2021–2022. A, B) Capture sites and the number of mosquitoes (A) and bird samples (B) screened for SINV by reverse transcription PCR throughout the Netherlands. C) Bird ringing sites (n = 258) of the bird ringers (n = 148) screened for SINV neutralizing antibodies throughout the Netherlands. D) Overview of the serologic screening of horses throughout the Netherlands and birds in a 16-km radius of SINV findings. DR, Drenthe; FL, Flevoland; FR, Friesland; GD, Gelderland; GR, Groningen; LB, Limburg; NB, Noord-Brabant; NH, Noord-Holland; OV Overijssel; SINV, Sindbis virus; UT, Utrecht; ZH, Zuid-Holland; ZL, Zeeland.

We used real-time reverse transcription PCR (RT-PCR) to test all mosquitoes and birds for SINV RNA (Appendix). All mosquito pools tested negative (Figure, panel A; Appendix Figure 2, panel A). Of the live bird samples screened (9,599 birds caught/tested once and 593 birds caught/tested repeatedly), we detected SINV RNA (pooled from swab and feather samples) in 1 adult European robin (*Erithacus rubecula*) caught in Naarden, Noord-Holland (October 23, 2022) (Figure, panel B; Appendix Figure 2, panels B, C). A partial SINV sequence from that bird clustered with SINV genotype I sequences from Germany, Nordic countries (Finland, Sweden, and Norway), and Russia (99% identity to all) (Appendix Figure 3). The partial SINV sequence was deposited in GenBank (accession no. PQ215107).

We initially tested a total of 368 horses for SINV neutralizing antibodies by using a plaque reduction neutralization test (PRNT) based on >80% plaque reduction (Appendix Figure 4) (*10*). Three (0.82%, 95% CI 0.28%–2.37%) of 368 mares sampled during July–December 2021 were seropositive, and endpoint titers ranged from 20 to 80 (Table; Figure, panel D). None of the horses traveled outside of the Netherlands. We examined serum samples of wild birds trapped within a 16-km radius of the 3 seropositive horses and the RT-PCR–positive bird by using PRNT (2021–2022; n = 110); 12 (10.91%, 95% CI 6.35%–18.10%) of those bird serum samples were seropositive (Table; Figure, panel D). Of the 110 bird samples tested, we derived 4 samples from 2 birds (identification no. L521438, positive and later negative; identification no. ID L413285, twice negative; Table). We found neutralizing antibodies in common blackbirds (*Turdus merula*; n = 10) and song thrushes (*Turdus philomelos*; n = 2) from the *Turdidae* family (Appendix Figure 5). The titers varied from 20 to 320 (Table). All the 148 human bird ringer serum samples tested were SINV seronegative (Figure, panel C). 

**Table:** Metrics of seropositive horses and birds and their neutralizing antibody titers as determined by PRNT_80_ for local circulation of Sindbis virus in wild birds and horses, the Netherlands, 2021–2022*

Site from Figure, panel D	Animal ID	Sampling date	Species, breed	Age, y/sex	PRNT_80_ titers	Clinical signs
1	Horse A	2021 Jul 31	Horse (*Equus caballus*), Dutch Warmblood	10/F	20	No
3	Horse B	2021 Oct 15	Horse, Friesian	1/F	80	Yes, neurologic
4	Horse C	2021 Sep 12	Horse, New Forest pony	30/F	20	No
4	L582243	2021 Nov 14	Common blackbird (*Turdus merula*)	>1/F	320	NA
3	L571081	2021 Sep 28	Common blackbird	1/M	320	NA
3	H363127	2022 Jun 22	Song thrush (*Turdus philomelos*)	>2/M	320	NA
3	L521438†	2021 Jun 21	Common blackbird	<1/M	40	NA
3	L521438†	2021 Oct 23	Common blackbird	<1/M	<20	NA
2	L586249	2022 May 1	Song thrush	>1/M	80	NA
3	L521227	2021 Jun 29	Common blackbird	4/F	20	NA
3	L571047	2021 Jun 30	Common blackbird	>2/M	20	NA
3	L521403	2021 Jan 6	Common blackbird	2/M	40	NA
3	L521265	2022 Oct 16	Common blackbird	1/M	160	NA
2	L586400	2022 Oct 23	Common blackbird	2/M	40	NA
1	L606047	2022 Dec 27	Common blackbird	>1/M	20	NA
1	L590812	2022 Oct 19	Common blackbird	>1/F	40	NA

*ID, identification; NA, not applicable; PRNT_80_, 80% plaque reduction neutralization test. †Same animal on 2 separate dates.

Our findings verify the circulation of SINV in the Netherlands by detection of RNA in a European robin and seropositive common blackbirds, song thrushes, and horses in distinct geographic regions. Seropositive birds caught in May and June (2 song thrushes and 3 blackbirds) and 1 bird caught multiple times within 1 season (1 blackbird) are considered resident breeding birds in the Netherlands and likely acquired an infection with SINV locally. The detection of neutralizing antibodies in horses without travel history and resident birds in the Netherlands does strongly indicate locally acquired infections. However, additional isolation of SINV (whole-genome) sequences from infected mosquitoes, animals, or humans in the Netherlands is required to make conclusions over the exact route of SINV introduction.

This study did not detect SINV seropositivity in humans, possibly because of the low sample size in this study. No human SINV infections have been reported thus far in the Netherlands. However, the lack of reported human SINV infections might be either caused by limited awareness and, therefore, no testing or absence of spillovers into the human population. Detailed studies are needed to assess possible human prevalence. Future studies should target screening of mosquitoes, birds, horses, and humans at the end of the mosquito season in the same areas where seropositive cases were detected thus far. Meanwhile, veterinarians and health practitioners in the Netherlands should increase their awareness regarding surveillance for SINV and potential risk to humans from its circulation.

Appendix Additional information for local circulation of Sindbis virus in wild birds and horses, the Netherlands, 2021–2022.
