# Supplementary material for: Local Circulation of Sindbis Virus in Wild Birds and Horses, the Netherlands, 2021–2022
Source: Emerg Infect Dis. 2025 Apr;31(4):863–6. doi: 10.3201/eid3104.241503 (PMC11950264; doi:10.3201/eid3104.241503)
Supplement: Appendix — Additional information for local circulation of Sindbis virus in wild birds and horses, the Netherlands, 2021–2022. [file 24-1503-Techapp-s1.pdf]

Article DOI: https://doi.org/10.3201/eid3104.241503

EID cannot ensure accessibility for supplementary materials supplied by authors. Readers who have difficulty accessing supplementary content should contact the authors for assistance.

Local Circulation of Sindbis Virus in Wild Birds and Horses, the Netherlands, 2021–2022

Appendix

Molecular Analysis

Before molecular analysis, mosquito samples were species determined and pooled either at Leiden University (2020) or at Centrum Monitoring Vectoren (CMV), Netherlands Food and Consumer Product Safety Authority (NVWA) (2021 and 2022) as described earlier (*1*). Subsequently, the mosquito pools were transported to Erasmus MC for molecular analyses. Morphologic identification of mosquitoes was performed as described by Becker et al. (*2*). The mosquitoes were pooled (maximum pool size was 10 mosquitoes) according to species level, sampling location, and time point (monospecific species pools belonging to the genera *Aedes*, *Anopheles*, *Coquillettidia*, *Culex,* and *Culiseta* and mixed pools with *Cx. pipiens*/*torrentium*). The pools were homogenized in 1 mL medium (DMEM, $NaHCO_3$, HEPES-buffered saline, penicillin/streptomycin, amphotericin). RNA was extracted from the homogenized mosquito pools by manual extraction with AMPure XP Beads (Beckman Coulter, Brea, CA). Wild bird screening for SINV RNA was performed on swabs (throat or cloacal) collected during the capturing and ringing of the birds. RNA was extracted from the bird swabs and feathers by using the Viral NA Large volume Kit and MagNA Pure 96 System (Roche Holding, Basel, Switzerland). A more detailed description of the wild bird and mosquito monitoring scheme are described elsewhere (E Münger et al., unpub. data, https://doi.org/10.1101/2024.12.16.628479).

The mosquito/wild bird eluate was tested by means of a SINV-specific real-time RT-PCR described by Sane et al. (*3*) (NS1: forward primer, GGTTCCTACCACAGCGACGAT; reverse primer, ATACTGGTGCTCGGAAAACATTCT; probe, TTGGACATAGGCAGCGCACCGG) by using the LightCyler 480 (Roche LifeScience,

Basel, Switzerland). Samples that were tested positive were confirmed in a second PCR targeting a different region of the genome (NS2: forward primer, CGTCGAAGACAGTAGATTCGGTTA; reverse primer, CGCGAACGCTTCGTCAA; probe, TCAACGGATGCCACAAAGCCGTAGAA), as well as being subjected to sequencing.

Partial sanger sequencing of SINV PCR products (forward primer: CATAACCCGTCGTCTAGC and reverse primer: TAGGCTGTTCTGGCACTT) was performed in addition to whole genome sequencing by using an amplicon-based Oxford Nanopore MinION (Oxford Nanopore technologies, https://nanoporetech.com) approach by using modified primers from Ling et al. (*4*) (172-bp fragment) and amplicon-based nanopore sequencing (245 bp fragment) from the open reading frame 1, NSP3 segment (GenBank accession no. PQ215107; Appendix Figure 3). An alignment was generated by using AliView (*5*), and 73 sequences were downloaded from BV-BRC (https://www.bv-brc.org) or the GenBank database (https://www.ncbi.nlm.nih.gov/genbank). From the alignment, a maximum-likelihood phylogenetic analysis was conducted by using the best-fitted nucleotide substitution model (GTR + F + I + G4) in the IQ-TREE web server (*6*). The resulting maximum-likelihood phylogenetic tree was visualized and edited with R 4.3.2 (*7*) by using the packages ggplot2 (v. 3.5.1) (*8)* and ggtree (v 3.10.1) (*9*) and revised by using Inkscape 1.3.2. The partial SINV sequence was shown to cluster with SINV genotype I sequences from Germany, Nordic countries (Finland, Sweden, and Norway), and Russia (99% identity to all). Virus cultivation of the SINV-positive isolate on mammalian (Vero ATCC, Vero E6) and mosquito (C6/36; *Aedes albopictus*) cells did not result in a CPE, and the supernatant was PCR negative 1, 2, 3, and 8 days after infection.

The robin was also trapped, ringed, and released 1 year before (October 31, 2021) at the same site but was not sampled.

References

1. Blom R, Schrama MJJ, Spitzen J, Weller BFM, van der Linden A, Sikkema RS, et al. Arbovirus persistence in North-Western Europe: are mosquitoes the only overwintering pathway? One Health. 2022;16:100467. PubMed https://doi.org/10.1016/j.onehlt.2022.100467

2. Becker N, Petrić D, Zgomba M, Boase C, Madon MB, Dahl C, et al. Mosquitoes: identification, ecology and control. Cham (Switzerland): Springer International Publishing; 2020. https://doi.org/10.1007/978-3-030-11623-1

3. Sane J, Kurkela S, Levanov L, Nikkari S, Vaheri A, Vapalahti O. Development and evaluation of a real-time RT-PCR assay for Sindbis virus detection. J Virol Methods. 2012;179:185–8. PubMed https://doi.org/10.1016/j.jviromet.2011.10.021

4. Ling J, Smura T, Lundström JO, Pettersson JH-O, Sironen T, Vapalahti O, et al. Introduction and dispersal of Sindbis virus from Central Africa to Europe. J Virol. 2019;93:e00620–19. PubMed https://doi.org/10.1128/JVI.00620-19

5. Larsson A. AliView: a fast and lightweight alignment viewer and editor for large datasets. Bioinformatics. 2014;30:3276–8. PubMed https://doi.org/10.1093/bioinformatics/btu531

6. Trifinopoulos J, Nguyen L-T, von Haeseler A, Minh BQ. W-IQ-TREE: a fast online phylogenetic tool for maximum likelihood analysis. Nucleic Acids Res. 2016;44(W1):W232-5. PubMed https://doi.org/10.1093/nar/gkw256

7. R Core Team. R: A language and environment for statistical computing [cited 2024 Dec 12]. https://www.R-project.org/

8. Wickham H. ggplot2: elegant graphics for data analysis, 2nd ed. Cham (Switzerland): Springer International Publishing; 2016. https://doi.org/10.1007/978-3-319-24277-4

9. Yu G. Using ggtree to visualize data on tree-like structures. Curr Protoc Bioinformatics. 2020;69:e96. PubMed https://doi.org/10.1002/cpbi.96

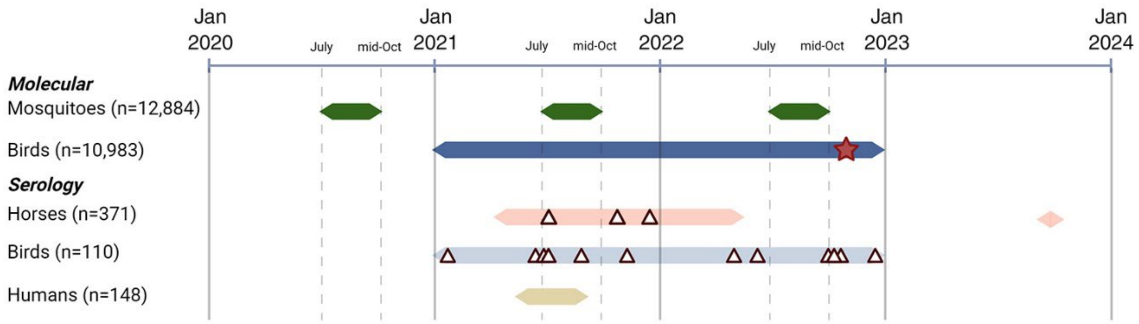

Appendix Figure 1. Timeline of sampling and Sindbis virus (SINV) findings per animal group. Red star (SINV-RNA) and triangles (SINV-antibodies) indicate positive samples. Figure created using BioRender (https://www.biorender.com).

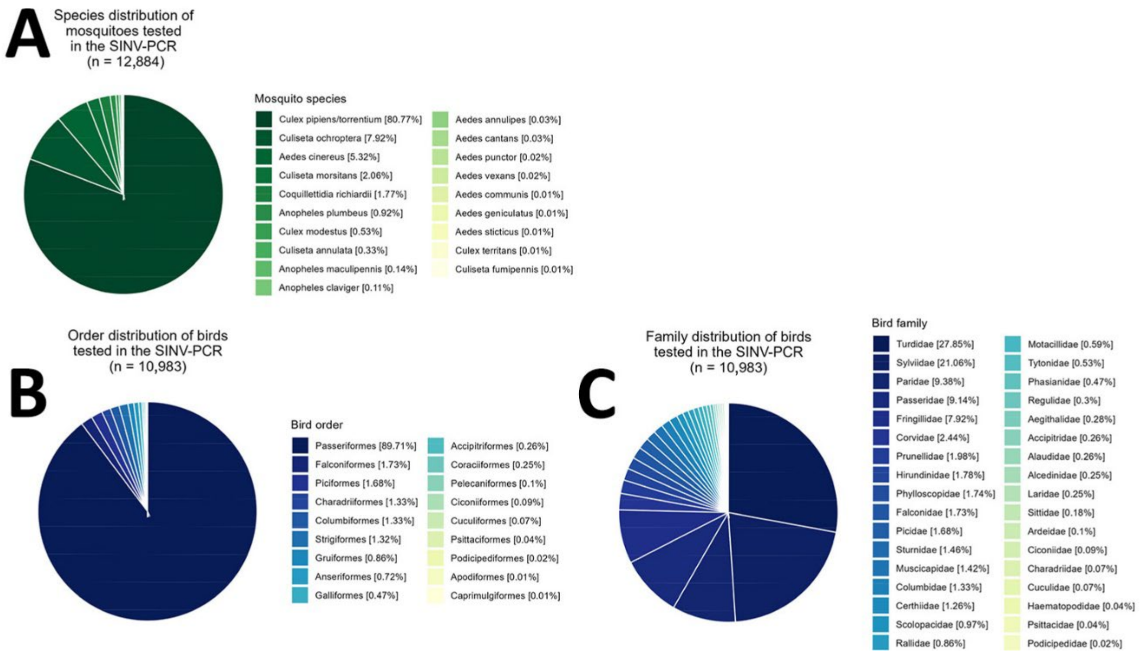

Appendix Figure 2. Pie charts portraying mosquito species distribution (A) and bird order (B) and family (C) distribution of samples screened for Sindbis virus by PCR.

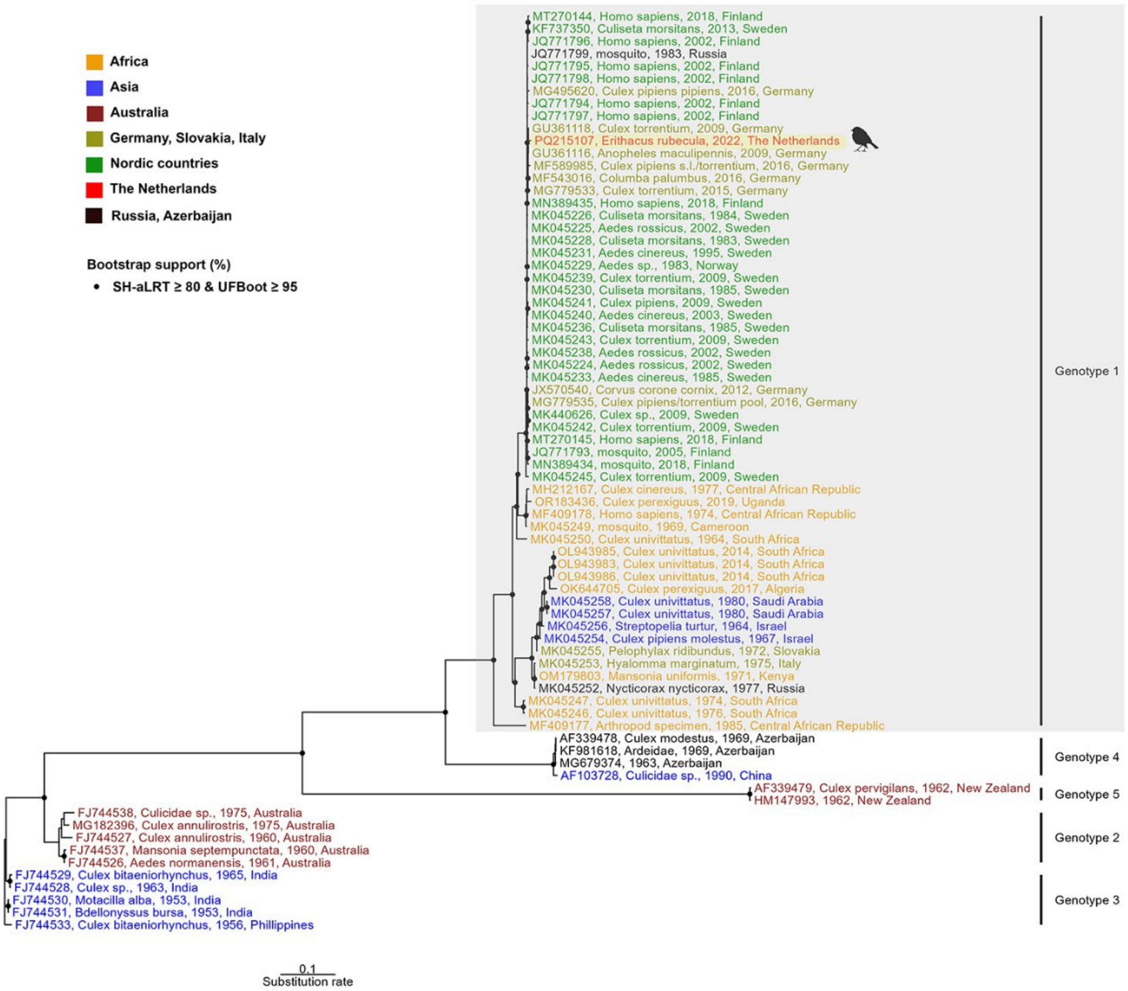

Appendix Figure 3. Phylogenetic tree based on the obtained partial SINV sequence highlighted in yellow (245 bp; region: open reading frame 1, nonstructural protein 3 [nsP3]) from the European robin from the Netherlands. Tip colors correspond to location of sequence and support values are represented on the respective branches by black circles when ultrafast bootstrap (UFBoot) ≥95% and SH-like approximate likelihood ratio test (SH-aLRT) ≥80%.

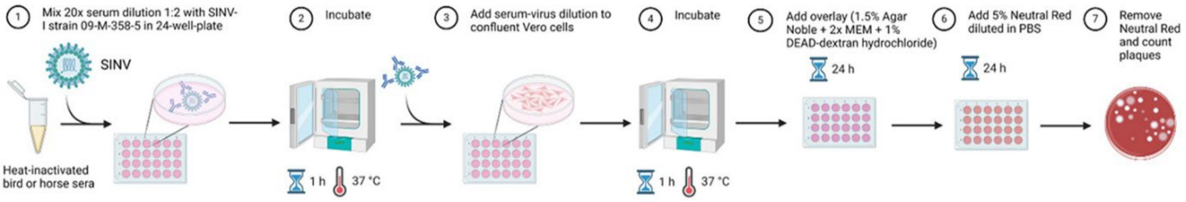

Appendix Figure 4. Workflow for testing horse and bird sera by means of a plaque reduction neutralization test (PRNT) for Sindbis virus (SINV). MEM, minimum essential medium; DEAD, diethylaminoethyl; PBS, phosphate-buffered saline. Figure created using BioRender (https://www.biorender.com).

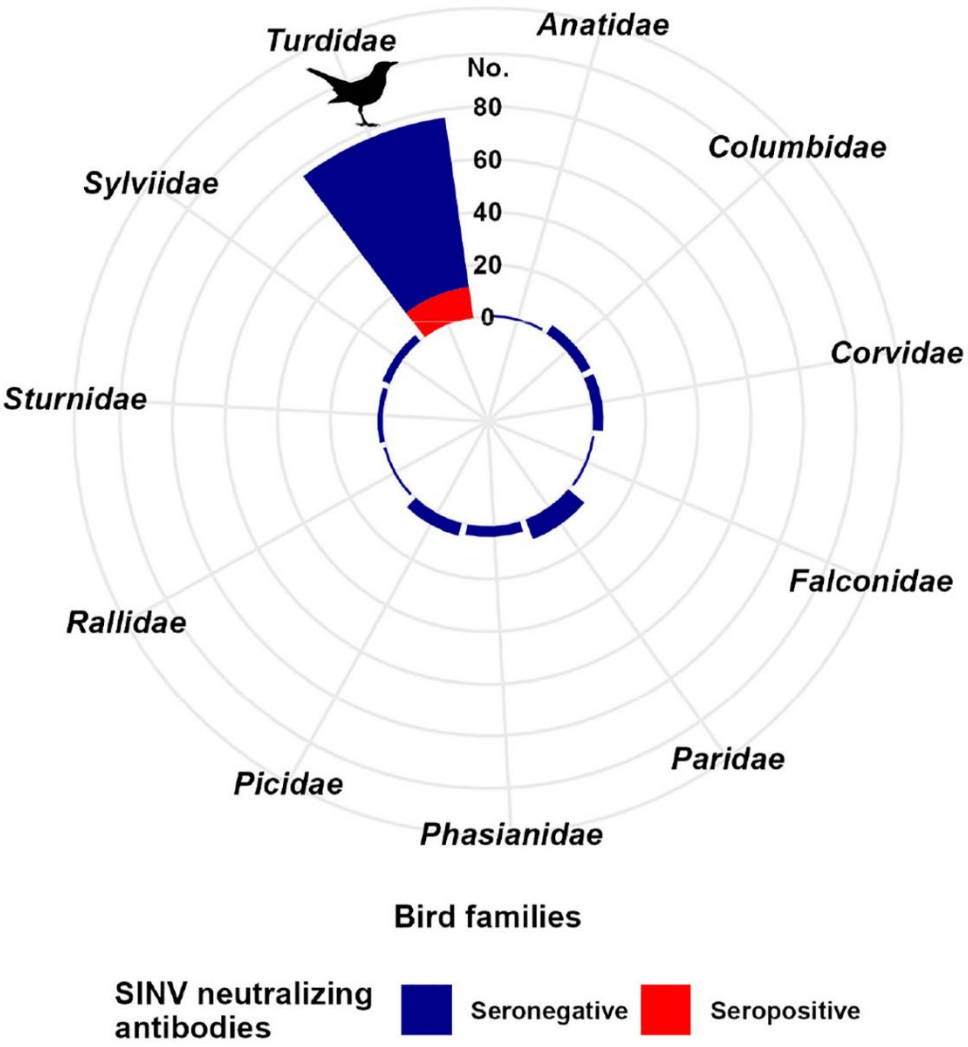

Appendix Figure 5. Circular bar chart showing family distribution of the bird samples tested and positive for SINV-neutralizing antibodies (n = 110).
